# Hepatitis B Vaccine-Associated Atypical Hemolytic Uremic Syndrome

**DOI:** 10.4274/Tjh-2013.0226

**Published:** 2013-12-05

**Authors:** Zekai Avcı, Cengiz Bayram, Barış Malbora

**Affiliations:** 1 Ankara Children’s Hematology and Oncology Research Hospital, Department of Hematology, Ankara, Turkey; 2 Ankara Pediatric and Pediatric Hematology Oncology Training and Research Hospital, Department of Pediatric Hematology, Ankara, Turkey; 3 Dr. Sami Ulus Research and Training Hospital of Women’s and Children’s Health and Diseases, Ankara, Turkey

**Keywords:** Hemolytic uremic syndrome, hepatitis B vaccine, children

## TO THE EDITOR

Hemolytic uremic syndrome (HUS) is one of the common causes of acute renal failure in children and is characterized by microangiopathic hemolytic anemia and thrombocytopenia. About 5% to 10% of all HUS cases in children are non-diarrheal HUS (atypical HUS) [1,2]. Many triggering causes of atypical HUS, such as non-enteric infections, viruses, drugs, systemic diseases, glomerulopathies, malignancies, transplantations, and pregnancy, have been identified [1,2,3,4]. Here we report a patient who developed atypical HUS after a hepatitis B vaccination. 

A 55-day-old female infant was admitted to our hospital with sudden onset of jaundice and pallor 1 day after the second dose of recombinant hepatitis B vaccine injection (containing 10 µg HBsAg/0.5 mL and 0.475 mg aluminum hydroxide/0.5 mL). There was no history of fever, diarrhea, or cough. The first dose of hepatitis B vaccine was administered at birth. She had no health problems in the neonatal period and was solely breastfed. The family history was non-contributory. 

On physical examination, the patient was in poor general condition and hypoactive with pale and icteric skin. Body temperature was 37 °C, pulse rate was 140/min, respiratory rate was 48/min, and blood pressure was 70/40 mmHg. There was no hepatosplenomegaly. Laboratory examination revealed a hemoglobin level of 51 g/L, leukocyte count of 10x109/L, platelet count of 28x109/L, and reticulocyte level of 3.9%. Anisocytosis, poikilocytosis, polychromasia, helmet cells, marked schistocytes, and rare platelets were observed in the peripheral blood smear, compatible with microangiopathic hemolytic anemia and thrombocytopenia. Direct and indirect Coombs test results were negative. Biochemical analyses were as follows: urea 88 mg/dL (normal range: 5-18), creatinine 1.1 mg/dL (normal: 0.2-0.4), total bilirubin 13.7 mg/dL (normal: <1.2), direct bilirubin 2.6 mg/dL (normal: <0.2), uric acid 8.1 mg/dL (normal: 2.4-6.4), aspartate aminotransferase 96 U/L (normal: 15-55), lactic dehydrogenase 4641 U/mL (normal: 170-580), and the other serum biochemical tests within normal limits. Urine microscopy showed numerous red blood cells. Microscopic examination of stool was normal and occult blood test results were negative. Her stool culture and urine culture were also negative. Serum complement component 3 (C3) and C4 levels were 76.5 mg/dL (normal: 79-752) and 5.89 mg/dL (normal: 16-38), respectively. Prothrombin time, activated partial thromboplastin time, and fibrinogen level were within the normal ranges. Renal ultrasound showed increased echogenicity in both kidneys. As the patient had acute renal failure, thrombocytopenia, and microangiopathic hemolytic anemia, she was diagnosed with HUS. After transfusion of red blood cells, intravenous fluid therapy was initiated and intravenous furosemide was administered. Fresh frozen plasma infusion was also started.

On the third day of hospitalization, she had a seizure that was ended with a single dose of midazolam and did not recur again. Hemolysis and thrombocytopenia continued until day 8 of hospitalization and the patient required red blood cell transfusions 5 times during this period. Urea and creatinine levels progressively rose to 119 mg/dL and 2.2 mg/dL, respectively. She had no oliguria. Hypertension was treated with nifedipine and enalapril. On day 9 of hospitalization, renal function, thrombocytopenia, and hemolysis began to improve, and plasma therapy was discontinued within the following 2 days. She no longer needed dialysis. The patient was discharged in good general condition on day 16 of hospitalization with improved complete blood count, biochemical tests, and complement levels. Factor H and factor I levels were normal when measured 3 months after hospital discharge. At 6 months of age, the patient’s hepatitis B antibody titer was at the protective level, and thus the third dose of hepatitis B vaccine was not administered. The patient completed the immunization schedule except for the third dose of hepatitis B without further problems. She is currently 38 months old and has no problems. 

Differentiation of HUS into typical HUS and atypical HUS may become confusing according to prodromal symptoms. Because infants below 6 months of age are generally breastfed (pre-weaning period), exposure to Escherichia coli O157:H7 is less likely in this age group and, therefore, other causes of HUS should be considered in patients under 6 months of age [4]. Our patient was a 55-day-old infant with no history of bloody diarrhea or other infection. Her stool and urine cultures were negative for Escherichia coli and Shigella. Her clinical presentation was consistent with the diagnosis of atypical HUS.

Our patient had clinical and laboratory findings of atypical HUS approximately 1 day after hepatitis B vaccine injection, and thus we suggest that the hepatitis B vaccine may play a triggering role for the onset of atypical HUS. Geerdink et al. [5] first reported a patient who developed atypical HUS a few days after a hepatitis B vaccination in their cohort study and they observed a relapse shortly after combined anti-diphtheria–pertussis–tetanus–polio vaccination in the same patient. To our knowledge, ours is the second case of atypical HUS associated with hepatitis B vaccination. In contrast to the first reported case, we did not observe a relapse with other vaccinations.

The triggering role of vaccination in the onset or relapse of atypical HUS has not been defined yet. We suggest that, compatible with the other adverse effects of vaccination, the immune-mediated activation of the complementary system triggers atypical HUS development. Therefore, recent history of vaccination should be examined, especially in patients without any other triggering conditions. Further reports are needed to confirm this hypothesis.

**Authors’ Contributions**

All authors planned and performed the experiments and wrote the manuscript. 

## CONFLICT OF INTEREST DISCLOSURE

There is no potential conflict of interest to disclose. 
